# Lack of association between TRPV1 gene polymorphisms and risk of migraine chronification: a case-control study and meta-analysis

**DOI:** 10.1007/s10072-024-07724-0

**Published:** 2024-08-06

**Authors:** Martina Giacon, Sarah Cargnin, Marta Allena, Rosaria Greco, Anna Maria Zanaboni, Sara Facchetti, Roberto De Icco, Grazia Sances, Natascia Ghiotto, Elena Guaschino, Daniele Martinelli, Cristina Tassorelli, Salvatore Terrazzino

**Affiliations:** 1 Department of Pharmaceutical Sciences, University of Piemonte Orientale “A. Avogadro”, Largo Donegani 2, Novara, 28100 Italy; 2 Department of Health Sciences, Università del Piemonte Orientale (UPO), Novara, Italy; 3 Headache Science and Neurorehabilitation Centre, IRCCS Mondino Foundation, Via Mondino, 2, Pavia, 27100 Italy; 4 Department of Brain and Behavioral Sciences, University of Pavia, Via Bassi 21, Pavia, 27100 Italy

**Keywords:** Episodic migraine, Chronic migraine, TRPV1, polymorphism, Meta-analysis

## Abstract

**Objective:**

To confirm a previously reported association of TRPV1 rs8065080 with the risk of transformation from episodic (EM) to chronic migraine (CM) and to extend knowledge about the role of other TRPV1 single nucleotide polymorphisms (SNPs), we first investigated the impact of three TRPV1 SNPs (rs8065080, rs222747 and rs222749) on the risk of migraine chronification in a case-control study. A systematic review and meta-analysis were then conducted to summarize the accumulated findings.

**Methods:**

Genotyping of the selected TRPV1 SNPs was performed using TaqMan real-time PCR in 167 EM and 182 CM participants. Crude and adjusted odds ratios with associated 95% confidence intervals were calculated in the log-additive, dominant, and recessive genetic models. A comprehensive literature search was performed in PubMed, Web of Knowledge, Cochrane Library, and OpenGrey until February 2024.

**Results:**

In our case-control study, no association was found between TRPV1 SNPs and the risk of migraine chronification, both in the unadjusted logistic regression models and after adjustment for confounding clinical variables. The results of the meta-analysis with a total of 241 participants with EM and 223 with CM confirmed no association between TRPV1 SNPs and the risk of migraine chronification in any of the genetic models tested.

**Conclusion:**

The results of the present case-control study and meta-analysis exclude a major role of TRPV1 rs8065080, rs222747, and rs222749 as risk factors for migraine chronification. However, further research is needed to investigate the gene-gene and gene-environment interactions of TRPV1 SNPs on the risk of transformation from episodic to chronic migraine.

**Supplementary Information:**

The online version contains supplementary material available at 10.1007/s10072-024-07724-0.

## Introduction

Migraine is a common neurovascular condition that affects around 15% of the global population [1]. It manifests itself in the form of recurrent attacks of moderate-to-severe pulsating head pain, which usually occurs on one side and is often accompanied by photophobia and phonophobia, nausea and vomiting. More than a third of people with migraine experience aura symptoms, which entail fully reversible neurological disturbances and occur early in migraine attack [2]. According to the International Classification of Headache Disorders (ICHD-III and 4 alpha), migraine is categorized as episodic (EM) if the attacks occur with or without aura for up to 14 days per month over the last 3 months [3–5]. In contrast, individuals are diagnosed with chronic migraine (CM) if they experience 15 or more headache days per month for more than three months, with at least 8 days exhibiting the clinical features of migraine [3–5]. Epidemiological evidence suggests that, annually, around 3% of people with EM progress to CM. The shift from EM to CM typically occurs gradually and involves various risk factors. These include non-modifiable factors like age, gender, hormonal fluctuations, and ethnicity, as well as environmental factors such as stress, sleep-related disorders, weather changes, smells, dietary components, and sensory triggers [6, 7]. Nonetheless, the excessive use of pain-relieving medications is now recognised as one of the most relevant factors triggering migraine chronification [8].

Significant efforts have been directed towards exploring the genetic basis of EM. The combined results of the two most recent and powerful migraine genome-wide association studies have identified a total of 178 independent single nucleotide (SNPs), of which LRP1 rs11172113, PRDM16 rs10218452 and FHL5 rs11153082 are the three most significantly associated SNPs [9]. However, our comprehension of the genetic factors driving the transition from EM to CM remains largely insufficient. The transient receptor potential (TRP) family encodes integral membrane proteins that act as ion channels in response to a variety of physical and chemical stimuli, including temperature, stretch/pressure, chemicals, oxidation/reduction, osmolarity, and pH [10]. Many TRP channels are expressed in nociceptive sensory neurons and are involved in pain generation and transmission [11]. Therefore, it has been postulated that TRP channels may play a role in the pathophysiology of migraine [10, 11]. Notably, a recent study published by Yakubova and colleagues in 2021 [12] reported a statistically different distribution of TRPV1 rs8065080 genotypes between EM and CM, suggesting a possible role of rs8065080 as a risk factor for migraine chronification. It is noteworthy that the TRPV1 gene encodes transient receptor potential vanilloid type 1 ion channels, which are expressed in trigeminal nociceptors and whose activation leads to the release of calcitonin gene-related peptide (CGRP), which is now recognized as a primary mediator of migraine pain [13]. The TRPV1 rs8065080 functional variant consists of an Ile585Val amino acid substitution (I585V), which leads to impaired TRPV1 receptor function in individuals expressing valine at position 585 [14]. This genetic variant has been extensively studied as a potential predictor of pain perception [15–19] along with two other single nucleotide polymorphisms (SNPs) affecting TRPV1 structural domains, namely rs222747 (I315M) and rs222749 (P91S) [15, 17, 20–22].

In the present study, we aimed not only to confirm the previous association of TRPV1 rs8065080 with migraine chronification but also to extend the knowledge about the role of other TRPV1 SNPs in the transformation of EM to CM. To this end, we first investigated the association of rs8065080, rs222747, and rs222749 of the TRPV1 gene with migraine chronification in a case-control study of participants with EM and CM. We then conducted a comprehensive systematic review of the literature followed by a meta-analysis to summarize the current evidence on the association between the above TRPV1 SNPs and the risk of migraine chronification.

## Methods

### Case-control study

A total of 349 Caucasian participants with migraine were prospectively and consecutively recruited by the Headache Science Centre of Mondino Institute of Pavia starting in 2018. The inclusion criteria for enrolment of subjects with EM were as follows: (1) age ≥ 18 years; (2) diagnosis of migraine fulfilling International Classification of Headache Disorders (ICHD)-III criteria; (3) less than 15 monthly headache days and, 4) duration of illness > 10 years. The requirement of the long duration of illness in the episodic form was adopted to minimize the likelihood of including people with EM that might have transitioned to CM in the future. The exclusion criteria were a previous or current diagnosis of medication overuse (MO) according to the ICHD-III. The inclusion criteria for enrollment of subjects with CM were the following: (1) age ≥ 18 years; (2) diagnosis of CM fulfilling International Classification of Headache Disorders (ICHD)-III criteria; and (3) duration of illness less than 5 years. This choice was made to avoid confounding variables associated with the consequences of the chronification rather than the cause. The study was performed in accordance with the guidelines of the Declaration of Helsinki. Authors obtained local ethics committee (N°20180034973) approval of the protocol. A written informed consent was obtained from all participants before their inclusion in the study.

### Genotyping

Genomic DNA was extracted from peripheral blood using the QiaAmp DNA Mini Kit (Qiagen Milan, Italy). We analysed three common TRPV1 SNPs (rs8065080, rs222747 and rs222749), which were selected based on their previous investigation in migraine and pain research [12, 15]. Genotyping of TRPV1 SNPs was performed by real-time polymerase chain reaction using Applied Biosystems TaqMan Pre-Designed SNP Genotyping assays (rs222749 Assay ID: C___1093692_30); rs222747 Assay ID: C___1093688_20; rs8065080 Assay ID: C__11679656_10), employing methods previously reported [23]. Real-time PCR amplification of TRPV1 SNPs was performed on a CFX Connect Real–Time PCR Detection System (Bio-Rad, Milan, Italy), and data analysis was conducted with the Bio-Rad CFX Manager Software (Version 3.1). Real-time PCR assays for each SNP included negative and positive controls for quality purposes. Approximately 10% of the samples were re-genotyped for validation purposes, and no discrepancies in genotyping were found between the assays. Genotyping was performed blind to all clinical data.

### Statistical analysis of case-control study

Each polymorphism was tested for deviation from the Hardy-Weinberg equilibrium (HWE) by using the freely available calculator at https://accounts.smccd.edu/case/biol215/docs/HW_calculator.xls. A SNP was considered to deviate from HWE at a significance level of *P*-value < 0.05. Statistical differences among groups of clinical variables were examined with the Student *t* test in the case of continuous variables with equal variances, or with the Welch *F* test for those with unequal variances. The χ2 test was used to assess differences between groups in the distribution of categorical variables. As regard the association of SNPs, the crude (i.e., unadjusted) odds ratio (OR) and the associated 95% confidence interval (CI) were calculated in the log-additive, dominant, and recessive genetic models. The log-additive genetic model is a trend test for the genotypes, similar to the allele model, but the comparisons are based on subjects (N) instead of the chromosomes (2 N). In the log-additive model, estimates are based on a logistic regression model in which genotypes are coded as 0, 1, or 2 to reflect the number of minor alleles. Adjusted ORs were calculated after adjusting for potential clinical confounders (i.e., clinical variables with a *P*-value < 0.05 in the respective univariate analysis). The crude and adjusted logistic regression analyses were performed using the MedCalc software. In all analyses, a two-tailed *P*-value < 0.05 was considered statistically significant.

### Systematic review

A comprehensive search of electronic databases (PubMed, Web of Knowledge, Cochrane Library, and OpenGrey) was conducted up until February 26th, 2024, to identify potentially eligible studies. A Boolean combination of the following keywords was used: (transient receptor potential OR TRPV1 OR GWAS) AND chronic migraine. Eligible studies were required to meet the following inclusion criteria: (1) studies comparing the genotype distribution of TRPV1 SNPs between people with EM and CM, with or without medication overuse; (2) studies investigating at least one of the following TRPV1 SNPs: rs222747, rs222749, and rs8065080. The following exclusion criteria were applied: (1) not human studies; (2) studies not related to the research topics; (3) reports, case series, meeting abstracts, editorials, letters to the editor, review articles, and meta-analyses; (4) studies not evaluating the association between TRPV1 polymorphisms and the risk of the transformation of EM into CM; (5) articles written in languages other than English. All potentially relevant studies identified in the first screening step were then read in full to determine whether they were eligible for inclusion in the study. A manual review of primary and review article references was also performed to identify additional relevant studies that had been overlooked in the initial electronic search. If relevant data could not be extracted from an eligible study, the corresponding author was contacted by email to request the missing information. The study was excluded from the systematic review and meta-analysis if the corresponding author did not respond to the email or did not provide the data required to calculate the effect size.

### Assessment of study quality

The quality of studies included in the systematic review was assessed using the Methodological Index for Non-Randomized Studies (MINORS criteria) [24], which comprises 8 items for non-comparative studies and 12 items for comparative studies, with a maximum score of 2 for each item. For comparative studies, a total score of less than 14 is considered poor quality, 15–19 is considered moderate quality, and 20–24 is considered good quality, with 24 being the ideal total score for comparative studies.

### Data extraction and meta-analysis

The recorded information for each study includes the first author’s last name, year of publication, study location, ethnicity, number of participants with EM and CM, the TRPV1 SNP investigated, and method of genotyping. Genotype distributions among participants with EM and CM were extracted from each study, and the HWE *P*-value was calculated for each group by using the free calculator available at https://accounts.smccd.edu/case/biol215/docs/HW_calculator.xls. ORs for the allelic, dominant, and recessive models were calculated and combined using the random-effects model, which assumes that the true effect size may differ from study to study due to differences (heterogeneity) among studies [25]. Heterogeneity between studies was estimated using the chi-squared-based Cochran’s Q test, and its statistical significance was set at *P* < 0.10. Between-study heterogeneity was also estimated by the I^2^ statistic, with an I^2^ value > 50% indicating a high level of heterogeneity. The presence of publication bias was assessed graphically by drawing funnel plots and statistically by the Egger’s test when at least three studies were present in the pooled analysis. All calculations were performed using MetaGenyo (http://bioinfo.genyo.es/metagenyo/), and the significance threshold for pooled results was set at a *P*-value < 0.05.

## Results

### Case-control study

The study comprised a total of 349 participants, including 167 subjects with EM and 182 with CM. Participants with EM had a mean age of 38.7 ± 11.0 years and were female in 78.4% (*n* = 131) of cases, while participants with CM had a mean age of 47.7 ± 10.9 years and were female in 85.2% (*n* = 155) of cases. The demographic and clinical characteristics of the two groups are shown in Table 1. Several clinical variables were statistically different between EM and CM groups (Table 1), including age, concomitant tension-type headache (TTH), caffeine consumption, alcohol consumption, smoking, snoring, insomnia, physical activity, use of anti-hypertensive drugs, antidepressant drug use, and use of benzodiazepines/antipsychotic drugs (all *P* < 0.05). In the vast majority of our CM participants (181 out of 182), chronic migraine is associated with the overuse of acute medications, with a mean number of monthly days with drug overuse of 23.4 (SD = 6.3). All three TRPV1 SNPs were found in HWE in both groups of subjects (all *P*-values > 0.05, Table 2). None of the selected TRPV1 SNPs was found associated with the risk of migraine chronification, both in the unadjusted logistic regression models and after adjustment for confounding clinical variables, as shown in Table 3.

**Table 1:** Comparison of clinical variables between participants with episodic migraine (EM, *n* = 167) and with chronic migraine (CM, *n* = 182)

Clinical variable	EM pts, *n*%	CM pts, *n*%	*P*-value
**Age** (*n* = 349), years mean (SD)	38.7 (11.0)	47.7 (10.9)	**< 0.0001**
**Migraine onset** (*n* = 349), years mean (SD)	15.7 (8.0)	15.2 (8.1)	0.611
**Gender**, (*n* = 349)			0.103
Female Male	131 (78.4) 36 (21.6)	155 (85.2) 27 (14.8)	
**Aura**, (*n* = 349)			0.442
No Yes	112 (67.1) 55 (32.9)	129 (70.9) 53 (29.1)	
**TTH**, (*n* = 348)			**0.011**
No Yes	148 (88.6) 19 (11.4)	142 (78.5) 39 (21.5)	
**Caffeine**, (*n* = 344)			**< 0.0001**
No Yes	85 (51.8) 79 (48.2)	34 (18.9) 146 (81.1)	
**Alcohol**, (*n* = 346)			**< 0.0001**
No Yes	116 (70.7) 48 (29.3)	89 (48.9) 93 (51.1)	
**Smoking**, (*n* = 346)			**0.0007**
No Present Past	131 (79.9) 28 (17.1) 5 (3.0)	124 (68.1) 31 (17.1) 27 (14.8)	
**Physical activity**, (*n* = 348)			**0.020**
No Yes	93 (56.0) 73 (44.0)	124 (68.1) 58 (31.9)	
**Snoring**, (*n* = 347)			**< 0.0001**
No Yes	146 (88.5) 19 (11.5)	107 (58.8) 75 (41.2)	
**Insomnia**, (*n* = 347)			**< 0.0001**
No Yes	118 (71.5) 47 (28.5)	84 (46.2) 98 ((53.8)	
**Antihypertensive drugs**, (*n* = 347)			**< 0.0001**
No Yes	157 (95.2) 8 (4.8)	141 (77.5) 41 (22.5)	
**Antidepressant drugs**, (*n* = 349)			**< 0.0001**
No Yes	159 (95.2) 8 (4.8)	118 (64.8) 64 (35.2)	
**Benzodiazepines or antipsychotics**, (*n* = 349)			**< 0.0001**
No Yes	154 (92.2) 13 (7.8)	126 (69.2) 56 (30.8)	

CM, chronic migraine; EM, episodic migraine; Pts, patients; SD, standard deviation; TTH, tension-type headache. Variables with *p* < 0.05 are in bold

**Table 2:** Main characteristics of studies included in the systematic review

First Author (Year)	Country	Ethnicity	EM/CM, *n*	TRPV1 Gene variant	Genotyping method	HWE *P*-value EM/CM
Ishibashi M (2018)	Japan	Japanese	47/22	rs222747, rs222749 and rs8065080	PCR-RFLP	rs222747: 0.11/0.09 rs222749: 0.32/0.70 rs8065080: 0.23/0.82
Yakubova A (2021)	Russia	Caucasian	27/19	rs8065080	AS-PCR	0.38/0.41
The present study	Italy	Caucasian	167/182	rs222747, rs222749 and rs8065080	TaqMan Real-time PCR	rs222747: 0.66/0.52 rs222749: 0.46/0.62 rs8065080: 0.68/0.65

AS-PCR, allele-specific polymerase chain reaction; CM, participants with chronic migraine; HWE, Hardy–Weinberg equilibrium; EM, participants with episodic migraine;

PCR-RFLP, polymerase chain reaction-restriction fragment length polymorphism

**Table 3:** Association analysis between TRPV1 SNPs and risk of migraine chronification in the present study

SNP	EM pts, *n* (%)	CM pts, *n* (%)	Crude OR (95%CI)	*P*-value	Adjusted OR* (95%CI)	*P*-value
**TRPV1 rs8065080**						
T/T T/C C/C	58 (34.7) 83 (49.7) 26 (15.6)	57 (31.3) 87 (47.8) 38 (20.9)	A: 1.19 (0.88–1.61) D: 1.17 (0.75–1.82) R: 1.43 (0.83–2.48)	0.259 0.486 0.203	A: 0.95 (0.63–1.43) D: 0.84 (0.46–1.54) R: 1.10 (0.53–2.31)	0.813 0.576 0.797
**TRPV1 rs222747**						
G/G G/C C/C	88 (52.7) 68 (40.7) 11 (6.6)	104 (57.1) 65 (35.7) 13 (7.1)	A: 0.90 (0.65–1.27) D: 0.84 (0.55–1.27) R: 1.09 (0.47–2.51)	0.549 0.408 0.840	A: 0.85 (0.53–1.36) D: 0.77 (0.43–1.36) R: 1.09 (0.33–3.59)	0.490 0.361 0.884
**TRPV1 rs222749**						
G/G G/A	148 (89.2) 18 (10.8)	169 (92.9) 13 (7.1)	1 (Ref) 0.63 (0.30–1.33)	0.226	1 (Ref) 0.92 (0.34–2.48)	0.871

*Adjusted by clinical variables found significant in the univariate analysis (i.e. age, alcohol consumption, physical activity, concomitant tension-type headache, caffeine use, smoking, insomnia, snoring, use of antihypertensive drugs, use of antidepressants drugs, use of benzodiazepines or antipsychotics)

A: Additive model, CM, chronic migraine; D: dominant model; EM, episodic migraine; Pts, patients; R: recessive model

### Systematic review

**Fig. 1:**
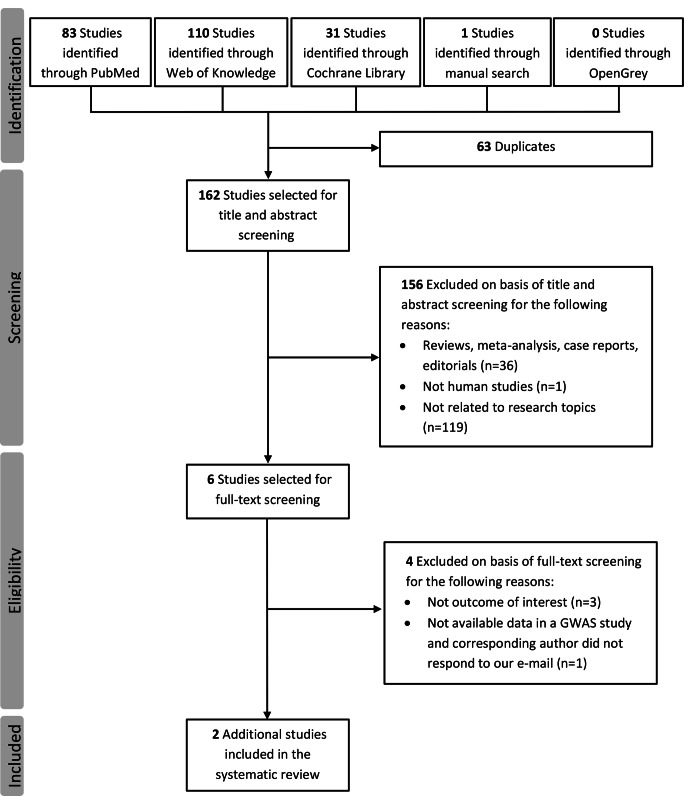
Flowchart of the literature search and selection process for eligible studies

A total of 225 potentially relevant studies were identified from databases (PubMed, *n* = 83; Web of Knowledge, *n* = 110; Cochrane Library, *n* = 31; OpenGrey, *n* = 0) and from manual searches of the literature (*n* = 1). After excluding 63 duplicates and applying inclusion and exclusion criteria to titles and abstracts, six studies were eligible for full-text review and assessment. After excluding 4 additional reports, 2 studies met the criteria for inclusion in the systematic review and meta-analysis [12, 26]. A detailed flowchart of the study selection process is shown in Fig. 1. It is noteworthy that the systematic review identified a genome-wide association study (GWAS) investigating genetic determinants of migraine progression to CM [27]. However, this study was excluded from the systematic review because the corresponding author was unavailable to provide the genotype distribution of the three TRPV1 SNPs in groups of individuals with EM and CM. The two studies identified in the systematic review were published in 2018 [26] and 2021 [12], with a total sample size of 69 and 46 subjects, respectively. The first published study [26] reported genotype data on three TRPV1 SNPs (rs8065080, rs222747, and rs222749), while the subsequently published study reported only the genotype distribution of TRPV1 rs8065080 [12]. The general characteristics of the three studies, including the present replication, are summarized in Table 2. The average percentage of MINORS score was 16.7 (range 13–22, Supplementary Table 1), indicating that the overall methodological quality was moderate.

### Meta-analysis

**Fig. 2:**
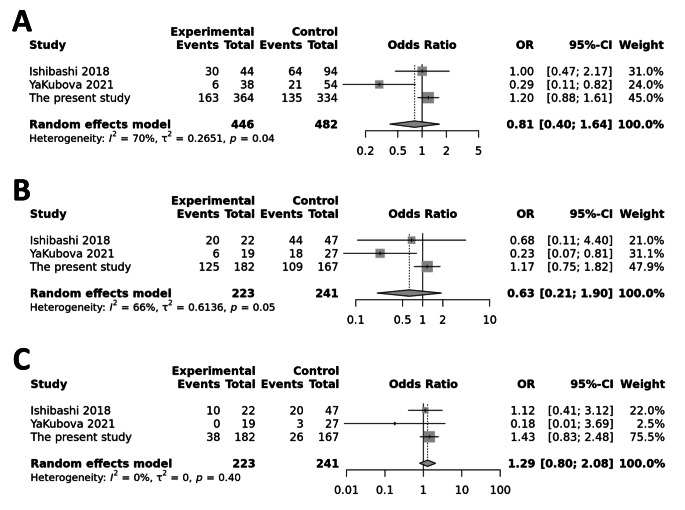
Forest plots for the association between TRPV1 rs8065080 and migraine chronification under the allelic (**A**), dominant (**B**), or recessive (**C**) genetic models of inheritance

Three studies involving a total of 464 participants (241 EM and 223 CM) were included in the meta-analysis of TRPV1 rs8065080 and the risk of migraine chronification. A significant association was found in the study by Yakubova et al. 2021 [12] for the allelic (C vs. T, Fig. 2A) and dominant (CC or CT vs. TT, Fig. 2B), but not for the recessive (CC vs. CT or TT, Fig. 2C) model of rs8065080. However, the pooled results showed no association in any of the three genetic models, in the presence of significant heterogeneity between studies for the allelic (I^2^ = 70%, *P* = 0.04) and dominant (I^2^ = 66%, *P* = 0.05) contrast of rs8065080. Funnel plots were carried out to evaluate the presence of publication bias (Supplementary Fig. 1A, B, C). The results indicated no evidence of significant publication bias in the results, as demonstrated by the Egger’s test (all *p*-values > 0.05).

In the meta-analysis of TRPV1 rs222747, two studies with a total sample size of 417 participants (214 EM and 204 CM) were available. Results of pooled ORs showed no significant impact of rs222747 under either the allelic (C vs. G, Supplementary Fig. 2A), dominant (CC or CG vs. GG, Supplementary Fig. 2B) or recessive (CC vs. CG or GG, Supplementary Fig. 2C) models. For the meta-analysis of rs222749, two studies were available for the allelic and dominant contrasts, while only one was available for the recessive model [26]. Results of the pooled analysis showed no association with the risk of migraine transformation for either the allelic (A vs. G, Supplementary Fig. 3A), dominant (AA or AG vs. GG, Supplementary Fig. 3B), or recessive (AA vs. AG or GG, Supplementary Fig. 3C) models of rs222749.

## Discussion

While a previous pilot study suggested a possible role of TRPV1 rs8065080 in the transformation from EM to CM [12], the present study found no association between rs8065080 and two other TRPV1 SNPs (rs222747 and rs222749) with the risk of migraine chronification. The results of the present case-control study were confirmed by the subsequent systematic review and meta-analysis, which combined our results with those from previously published studies investigating rs8065080, rs222747 and/or rs222749 of the TRPV1 gene as susceptibility factors for the transformation of migraine from the episodic to the chronic form.

The interest in investigating the involvement of TRPV1 channels in migraine stems primarily from their localization on meningeal nociceptors and their ability to respond to various endogenous and exogenous stimuli involved in migraine pathogenesis, including capsaicin, endocannabinoids, endovanilloids, prostaglandins, nerve growth factors, and lipoxygenase metabolites [28, 29]. TRPV1 is known to be expressed in approximately 10–20% of small and medium-sized neurons of the trigeminal ganglion [30], and CGRP has been reported to be co-expressed in a subset of TRPV1-positive neurons [31], particularly in the hippocampus, thalamus, basal ganglia, hypothalamus and the trigeminal fibers innervating the dura [32–34]. There is increasing evidence that cortical spreading depression may activate TRPV1 receptors, which in turn cause an increased release of CGRP from C-fibers, ultimately contributing to trigeminal sensitization and exacerbation of migraine attacks with associated symptoms [29, 35, 36]. On the other hand, immunohistochemical studies showed that TRPV1 was significantly more expressed on nerve fibers in the arterial walls of scalp vessels of people with CM compared to control subjects [37], suggesting that TRPV1 heightened expression may serve as a neurophysiological factor involved in CM pathophysiology. In addition, preclinical studies with repeated 30-day administration of eletriptan or indomethacin in rat models of CM have shown increased TRPV1 transcript levels in the trigeminal ganglia. It has been hypothesized that this change is triggered by the migraine itself and/or by migraine medications and ultimately leads to sensitization of the pronociceptors and exacerbation of migraine symptoms in people with CM [38].

Although the rationale for investigating TRPV1 as a central role in the pathophysiology of CM is robust, our results exclude a role of three extensively studied polymorphisms of the TRPV1 gene as risk factors for migraine chronification. On the other hand, clinical observations suggest that certain environmental factors may facilitate the transition of migraine from an episodic to a chronic type [7, 8]. The results of the present study showed several modifiable factors that were statistically different between EM and CM, including caffeine consumption, smoking habits, alcohol consumption, physical activity, and drug use. Although it can be argued that environmental factors rather than genetic factors may be involved in migraine chronification and that our study may have insufficient statistical power to detect gene variants with a small effect size, our study nevertheless rules out a clinically relevant effect of the investigated TRPV1 SNPs as risk factors for transformation of EM to CM.

We are aware of the limitations of this study that need to be considered for a correct interpretation of the results. First, the sample size of our case-control study was limited, so our study may be underpowered to detect gene polymorphisms with small effect sizes on the risk of migraine chronification. However, the results of our case-control study were confirmed by pooled estimates from the systematic review and meta-analysis. Second, only three common TRPV1 polymorphisms were selected for the case-control study, which were chosen based on their previous investigation in migraine research. Therefore, we cannot exclude the possibility that other TRPV1 SNPs, including polymorphisms with low frequency and large effect sizes, may contribute to the risk of EM transition to CM. Third, despite our attempt to perform a comprehensive analysis of all available published studies, we are unable to include data from a genome-wide association study (GWAS) on migraine chronification [27] because the corresponding author was unable to provide genotype data for the three TRPV1 SNPs studied. Finally, as we did not have access to the individual data of the primary studies, our meta-analysis relies on unadjusted risk estimates. Therefore, a meta-analysis of individual participant data should be performed to obtain pooled estimates for TRPV1 SNPs adjusted for confounding variables such as sex, age, lifestyle, and comorbidities.

In conclusion, the results of the present case-control study and meta-analysis exclude a role of TRPV1 rs8065080, rs222747 and rs222749 as risk factors for migraine chronification. However, due to the aforementioned limitations of the present study and the complex pathophysiology of CM, further studies, preferably with a multicenter design and larger sample size, are needed to explore the genetic architecture of migraine chronification and to investigate the gene-gene and gene-environment interactions of TRPV1 SNPs on the risk of transformation from EM to CM.

## Electronic supplementary material

Below is the link to the electronic supplementary material.

Supplementary Material 1
