# Supplementary material for: Lack of association between TRPV1 gene polymorphisms and risk of migraine chronification: a case-control study and meta-analysis
Source: Neurol Sci. 2024 Aug 6;46(1):303–12. doi: 10.1007/s10072-024-07724-0 (PMC11698790; doi:10.1007/s10072-024-07724-0)

LACK OF ASSOCIATION BETWEEN TRPV1 GENE POLYMORPHISMS AND RISK OF MIGRAINE CHRONIFICATION: A CASE-CONTROL STUDY AND META-ANALYSIS

NEUROLOGICAL SCIENCES

Martina Giacon[1], Sarah Cargnin[2], Marta Allena[3], Rosaria Greco[3], Anna Maria Zanaboni[3,4], Sara Facchetti[3,4], Roberto De Icco[3,4], Grazia Sances[3], Natascia Ghiotto[3], Elena Guaschino[3], Daniele Martinelli[3,4], Cristina Tassorelli[3,4] and Salvatore Terrazzino[1]

[1]Department of Pharmaceutical Sciences, University of Piemonte Orientale "A. Avogadro", Largo Donegani 2, 28100 Novara, Italy.

[2]Department of Health Sciences, Università del Piemonte Orientale (UPO), Novara, Italy.

[3] Headache Science and Neurorehabilitation Centre, IRCCS Mondino Foundation, Via Mondino, 2,

27100, Pavia, Italy.

[4]Department of Brain and Behavioral Sciences, University of Pavia, Via Bassi 21, 27100 Pavia, Italy.

Correspondence to:

Salvatore Terrazzino, PhD

Department of Pharmaceutical Sciences, University of Piemonte Orientale "A. Avogadro". Largo Donegani 2, 28100 Novara, Italy. Address email: salvatore.terrazzino@uniupo.it

Supplementary Table 1. Assessment of study quality by using the MINORS criteria.

#	*Methodological items for non-randomized studies*	*Score per item †*		
		**Ishibashi M et al., 2018**	**Yakubova A et al., 2021**	**The present study**
1	A clearly stated aim: the question addressed should be precise and relevant in the light of available literature	2	2	2
2	Inclusion of consecutive patients: all patients potentially fit for inclusion (satisfying the criteria for inclusion) have been included in the study during the study period (no exclusion or details about the reasons for exclusion)	2	0	2
3	Prospective collection of data: data were collected according to a protocol established before the beginning of the study	0	0	2
4	Endpoints appropriate to the aim of the study: unambiguous explanation of the criteria used to evaluate the main outcome which should be in accordance with the question addressed by the study. Also, the endpoints should be assessed on an intention-to-treat basis.	2	2	2
5	Unbiased assessment of the study endpoint: blind evaluation of objective endpoints and double-blind evaluation of subjective endpoints. Otherwise, the reasons for not blinding should be stated	0	2	2
6	Follow-up period appropriate to the aim of the study: the follow-up should be sufficiently long to allow the assessment of the main endpoint and possible adverse events	2	2	2
7	Loss to follow up less than 5%: all patients should be included in the follow up. Otherwise, the proportion lost to follow up should not exceed the proportion experiencing the major endpoint	2	2	2
8	Prospective calculation of the study size: information of the size of detectable difference of interest with a calculation of 95% confidence interval, according to the expected incidence of the outcome event, and information about the level for statistical significance and estimates of power when comparing the outcomes	0	0	0
9	An adequate control group: having a gold standard diagnostic test or therapeutic intervention recognized as the optimal intervention according to the available published data	2	2	2
10	Contemporary groups: control and studied group should be managed during the same time period (no historical comparison)	2	0	2
11	Baseline equivalence of groups: the groups should be similar regarding the criteria other than the studied endpoints. Absence of confounding factors that could bias the interpretation of the results	0	0	2
12	Adequate statistical analyses: whether the statistics were in accordance with the type of study with calculation of confidence intervals or relative risk	1	1	2
	Total score	15	13	22

† The items are scored 0 (not reported), 1 (reported but inadequate) or 2 (reported and adequate). The global ideal score for comparative studies comprising 12 items is 24.

Supplementary Fig. 1

Funnel plots for the association of TRPV1 rs8065080 with risk of migraine chronification. A. Allelic contrast: C vs. T, Egger's P-value = 0.366. B. Dominant contrast: CC+CT vs. TT, Egger's P-value = 0.446). C. Recessive contrast: CC vs. CT+TT, Egger's P-value = 0.113.

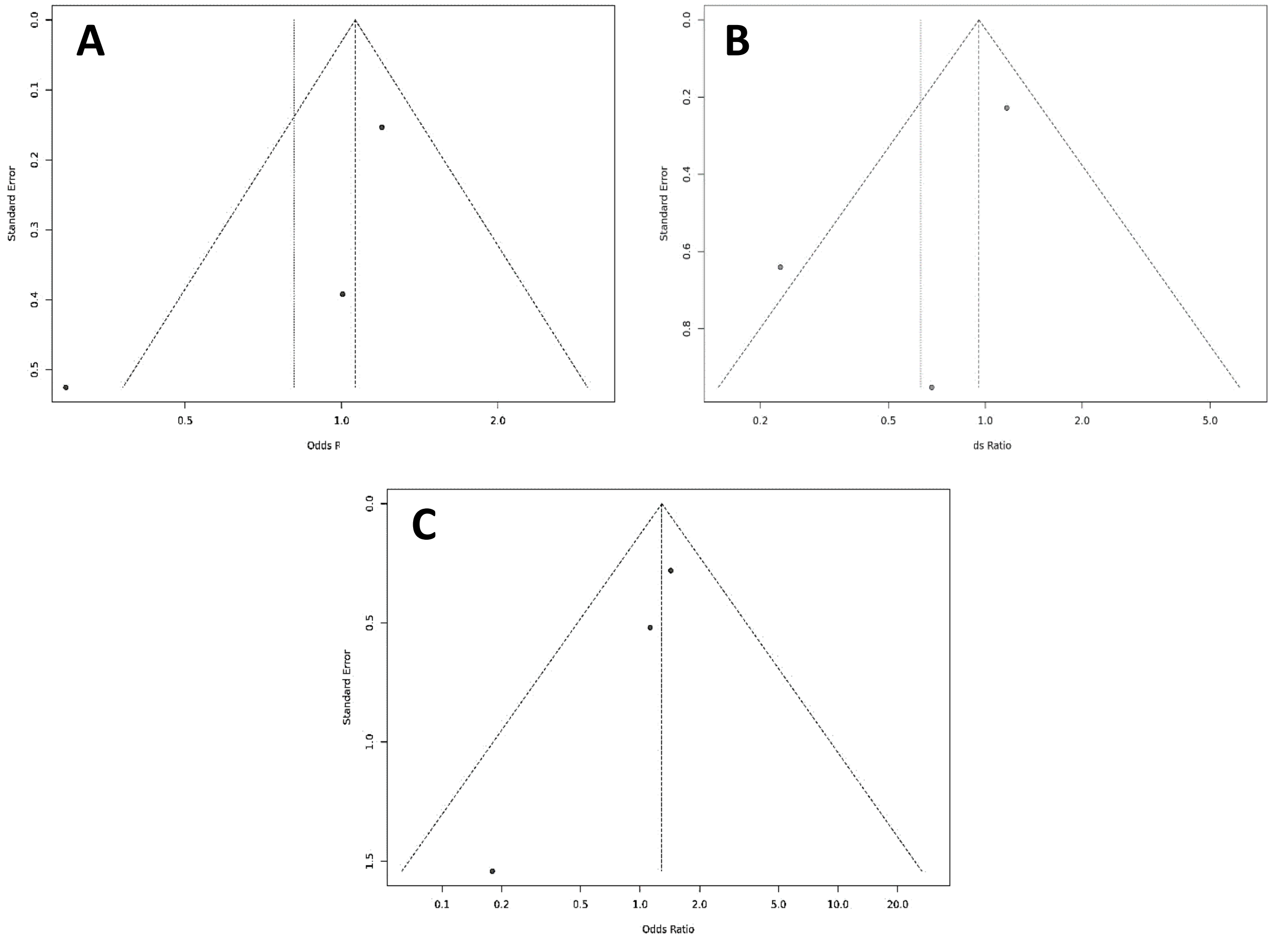

Supplementary Fig. 2

Forest plots for the association between TRPV1 rs222747 and migraine chronification under the allelic (A), dominant (B), or recessive (C) genetic models of inheritance.

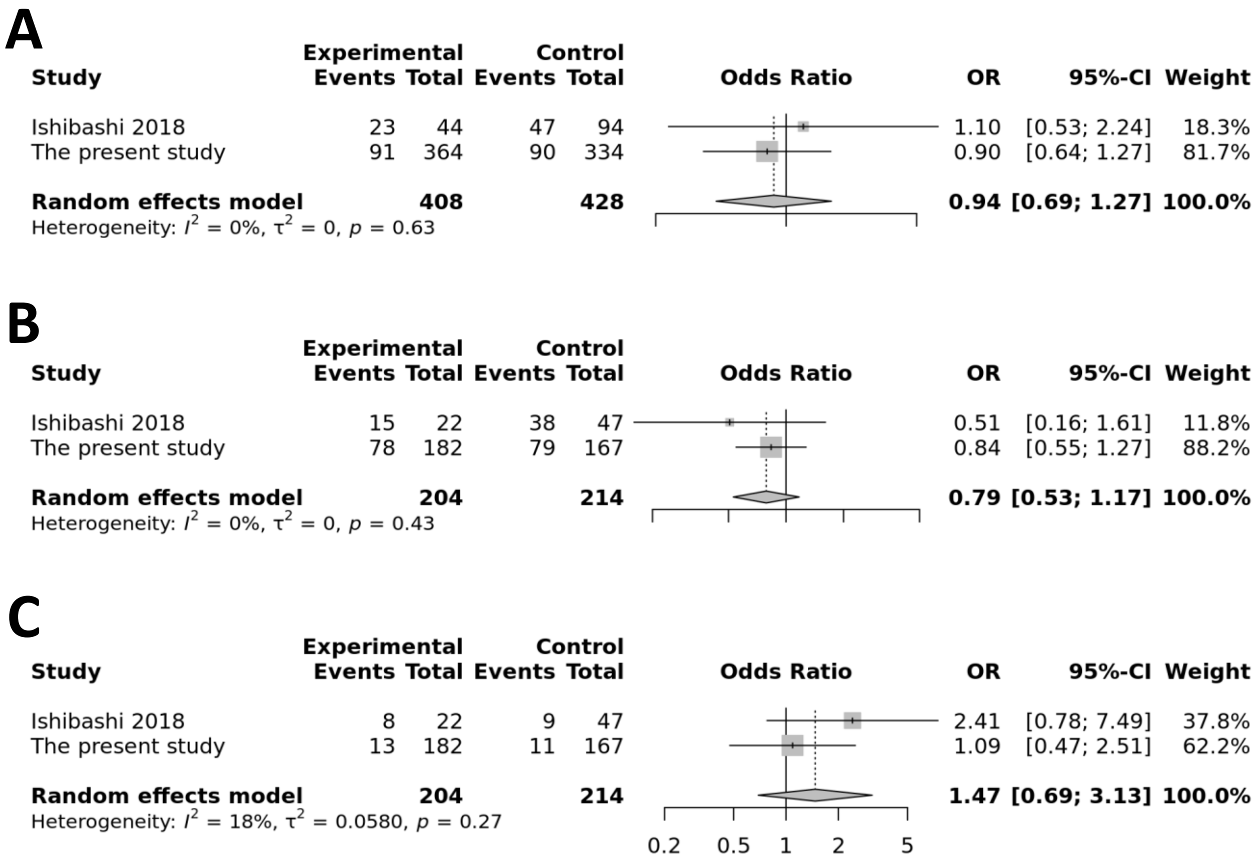

Supplementary Fig. 3

Forest plots for the association between TRPV1 rs222749 and migraine chronification under the allelic (A), dominant (B), or recessive (C) genetic models of inheritance.

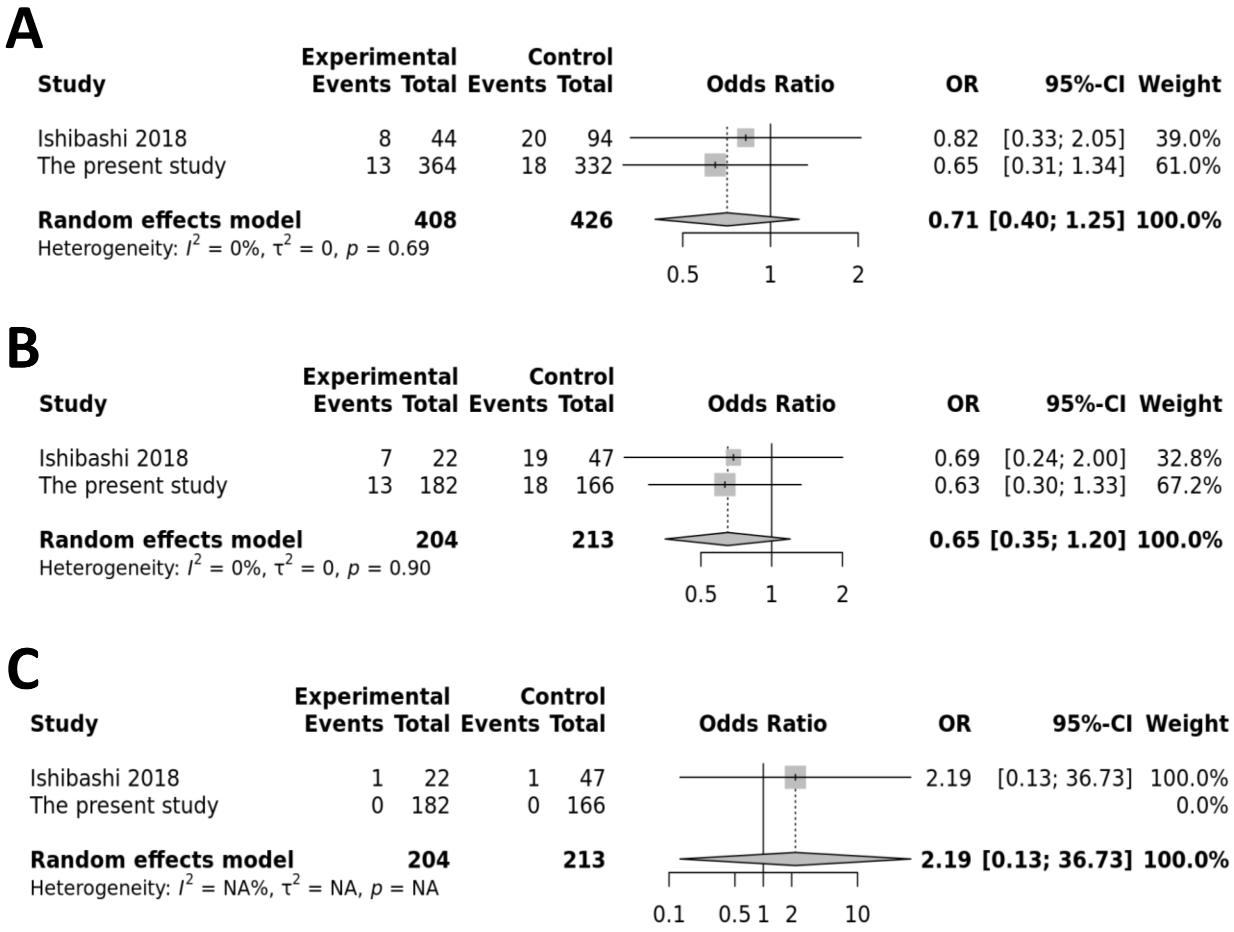

Supplement: Supplementary file 1 — Supplementary Material 1 [file 10072_2024_7724_MOESM1_ESM.pdf]
